# Taming Alzheimer’s disease: New perspectives, newer horizons

**Published:** 2017-07-06

**Authors:** Debraj Sen, Anusree Majumder, Vijinder Arora, Neha Yadu, Ritwik Chakrabarti

**Affiliations:** 1 Department of Radiology, Military Hospital, Jodhpur, India; 2 Department of Pathology, Military Hospital, Jodhpur, India; 3 Department of Radiology, Sri Guru Ramdas Institute of Medical Sciences and Research, Amritsar, India; 4 Department of Radiology, Command Hospital, Lucknow, India; 5 Department of Radiology, Military Hospital, Thiruvananthapuram, India

**Keywords:** Alzheimer Disease, Amyloid, Biomarkers, Cerebrospinal Fluid, Magnetic Resonance Imaging, Positron Emission Tomography

## Abstract

Alzheimer’s disease (AD) is the leading cause of dementia. However, current therapies do not prevent progression of the disease. New research into the pathogenesis of the disease has brought about a greater understanding of the “amyloid cascade” and associated receptor abnormalities, the role of genetic factors, and revealed that the disease process commences 10 to 20 years prior to the appearance of clinical signs. This greater understanding of the disease has prompted development of novel disease-modifying therapies (DMTs) which may prevent onset or delay progression of the disease. Using genetic biomarkers like apolipoprotein E (ApoE) ε4, biochemical biomarkers like cerebrospinal fluid (CSF) amyloid and tau proteins, and imaging biomarkers like magnetic resonance imaging (MRI) and positron emission tomography (PET), it is now possible to detect preclinical AD and also monitor its progression in asymptomatic people. These biomarkers can be used in the selection of high-risk populations for clinical trials and also to monitor the efficacy and side-effects of DMT. To validate and standardize these biomarkers and select the most reliable, repeatable, easily available, cost-effective and complementary options is the challenge ahead.

## Introduction

Progressively increasing life expectancy and declining fertility rates have led to a steady demographic shift towards the elderly population and consequently the prevalence of neurodegenerative diseases is on the rise. Alzheimer’s disease (AD) is the leading cause of dementia and about 0.5% of the global population or 35 million patients are currently afflicted worldwide.^1^^,^^2^ This number is expected to quadruple by the year 2050, with an attendant huge human and socioeconomic burden.^3^ Despite the disconcerting statistics, these are exciting times as we are at the cusp of better understanding of the disease with the promise of novel disease-modifying therapy (DMT). Simultaneously work is in progress on multi-target-directed ligands (MTDLs), hybrid drugs that act simultaneously on multiple targets, leading to greater therapeutic benefit and simplification of therapeutic regimens. 

This article though not exhaustive in scope or detail, outlines the purported pathogenesis of the disease, the ongoing research in novel DMT drugs targeting different sites, and the role of diagnostics in detecting the disease in a preclinical state, monitoring disease progress and their complementary role in developing DMT.

**Pathogenesis of AD**

AD is a multifactorial disease and knowledge of the pathogenesis of AD is essential for understanding the role of diagnostics in its detection, monitoring and development of novel DMT. AD is associated with regional cerebral hypometabolism, extracellular Aβ plaques, intracellular neurofibrillary tangles (NFTs) containing hyperphosphorylated tau, neuroinflammation and oxidative stress, loss of synaptic connections, neural death and atrophy and resultant clinical manifestations of AD.^4^^-^^7^ The progressive accumulation of Aβ and NFT is believed to begin more than 15 (Aβ) to 10 (NFT) years prior to onset of clinical disease.

Deposition of amyloid-β (Aβ) peptide in the brain is considered to represent the primary event in AD^8^ and the amyloid precursor protein (APP), a trans-membrane receptor, is central to the pathogenesis though its exact function is unknown. Aβ is generated by sequential proteolytic cleavage of APP by β-secretase or B-site APP cleaving enzyme (BACE)-1 from within and by γ-secretase from outside the membrane. When normally soluble Aβ peptides attain a definite level, they become insoluble, misfold and aggregate into Aβ plaques. These plaques are composed of insoluble peptides, generally 42 amino acids in length (Aβ_1-42_) and the oligomeric forms of Aβ_1-42_ are thought to have a greater neurotoxic potential than monomers or fibrils.^9^^,^^10^ Cleavage of APP by the α-secretase followed by γ-secretase generates neuroprotective amyloid precursor protein (APPα).^11^

Apolipoprotein E (ApoE) ε4 allele of ApoE gene encodes the transporter of cholesterol in the brain and is the major genetic susceptibility factor for late-onset AD.^12^^,^^13^ AD is associated with higher membrane-associated free cholesterol and overall greater brain cholesterol load. Genetic mutations of APP (chromosome 21) or trisomy 21 cause early onset autosomal dominant AD.^14^^-^^16^ Mutations of presenilin-1, presenilin-2, clusterin (CLU), phosphatidylinositol-binding clathrin assembly protein (PICALM), complement component (3b/4b) receptor 1 (CR1) and triggering receptor expressed on myeloid cells 2 (TREM2) have also been found to be associated with the disease.^17^^-^^20^ While increased production of Aβ may cause early-onset AD, late onset AD may be caused by impaired Aβ clearance due to interactions with ApoEε4, reduced proteolysis, decreased transport across the blood-brain barrier, or inefficient cerebrospinal fluid (CSF) transport.^21^

Tau binds and stabilizes microtubules and supports axonal transport. Tau interacts with Fyn in the postsynaptic compartment. Fyn phosphorylates the N-methyl-D-aspartate (NMDA) receptor subunit 2B (NR2B) and facilitates its interaction with postsynaptic density protein 95 (PSD95).^22^ The NR2B/PSD95 interaction is essential in Aβ-induced neurotoxicity.^23^ As increased neuronal membrane cholesterol is an important factor in AD and also causes overexpression of Fyn gene, this could be the link between cholesterol, Aβ and tau.^24^

Casoli, et al. have proposed that mitochondrial DNA mutations are also important factors in AD.^25^ The two structural proteins NEDD9 (neural precursor cell expressed, developmentally down-regulated 9) and CASS4 (Cas scaffolding protein family member 4) and the kinase PTK2B (protein tyrosine kinase type 2 beta) apart from their roles in neoplasia, have been found to have a role in inflammation, hypoxia, vascular changes, microtubule stability and calcium signaling, and are relevant in AD.^26^

Altered post-translational modification in the form of autophosphorylation of serine (Ser) and threonine (Thr), blocks the phosphorylation of tyrosine (Tyr) on insulin substrate receptor proteins (IRS) 1 and 2, impairs insulin signaling and leads to diabetes and AD-like complications. O-glycosylation of these Ser and Thr sites prevents phosphorylation and these sites may be targets for future drugs.^27^

Metal dyshomeostasis has also been proposed as a contributing factor to the disease. Herpes simplex virus-1 (HSV) has been investigated as a potential cause of AD with a role of antiviral therapy in future.^28^ Deficiency of nutritional factors like docosahexaenoic acid (DHA) and vitamins have also been implicated in the causation of AD and are being investigated.^29^ People with higher educational attainment or socioeconomic status have been found to have a greater reserve against AD as do people who exercise regularly.^30^^-^^32^

**Biomarkers**

The inaccessibility of the brain for histopathologic confirmation and the long preclinical phase, make surrogate markers of the disease that provide a biological measure of the ongoing disease, irrespective of symptomatology, very important. This is especially so with the advances in DMT and hence these have been incorporated into the new diagnostic criteria for AD (albeit for research purposes only). These biomarkers can be genetic (ApoE genotype), biochemical (CSF or plasma) or imaging biomarkers. They have a role not only in early diagnosis and prognosis, but also in selection of subjects for shorter and smaller clinical trials with greater statistical power (selection of inclusion and exclusion criteria), in providing evidence of target engagement and disease-modifying effects of DMT (as surrogate end points) and in monitoring side-effects of DMT. 

**CSF biomarkers**

Due to its direct contact with the brain extracellular space, CSF constituents closely reflect molecular events in the brain. Low CSF Aβ_1-42 _is a sensitive marker of cerebral Aβ deposition but it does not correlate well with duration or severity of disease. Low CSF Aβ is not very specific for AD as it is also seen in frontotemporal dementia (FTD), vascular dementia, Creutzfeldt–Jakob disease (CJD) and dementia with Lewy bodies (DLB). CSF tau (total-tau or t-tau and tau phosphorylated at threonine 181 or p-tau_181_) is increased in AD and its higher levels correlate with greater cognitive impairment. When Aβ_1-42 _and t-tau are considered together (Aβ_1-42 _to t-tau ratio), the sensitivity and specificity of diagnosing AD is more than 85%.^33^ Huded, et al. in a study from southern India also found that p-tau/t-tau and p-tau/Aβ ratio are good indicators of severity of dementia and may help differentiate between mild AD and moderate to severe AD.^34^ Different phosphorylated epitopes of tau may also be helpful in distinguishing AD and FTD (p-tau_231_) or AD and DLB (p-tau_181_).^35^^,^^36^

Levels of orexin (hypocretin), a neuropeptide that regulates arousal, wakefulness and appetite are altered in AD. Liguori, et al. have found that CSF orexin levels correlate with total tau protein levels, sleep impairment and cognitive decline in moderate to severe AD.^37^

**Imaging biomarkers**

The diagnostic value of imaging in AD is in identifying characteristic topographical, structural and functional alterations in the brain and in differentiating it from other causes of cognitive decline. 

**Volumetric magnetic resonance imaging (MRI)**

AD is characterized by progressive atrophy of the medial temporal lobe (MTL) in a typical sequence: entorhinal complex, followed by hippocampus, amygdala, parahippocampus and posterior cingulate gyrus. Patients with atypical language and visual presentations have left temporal and occipital atrophy, respectively. Volumetric MRI (T1-weighted imaging) has been validated against pathological post-mortem markers such as Braak stages and is the most mature imaging biomarker of disease progression.^38^ Savva, et al. in their epidemiological-autopsy study of individuals with and without dementia found that though plaques, tangles and atrophy were all associated with dementia, atrophy was most strongly related to dementia.^39^ Progression of whole cerebral and hippocampal atrophy closely matches clinical worsening in AD.^40^ Visual evaluation of MTL atrophy vis-à-vis normal ageing has a sensitivity and specificity of around 80%-85%. Paradoxical hippocampal volume loss noted after anti-amyloid immunotherapy is likely due to amyloid removal and fluid redistribution rather than atrophy. Strict standardization is required for manual volumetry and automated software like FreeSurfer, learning embeddings for atlas propagation (LEAP), and QUARC analysis software. Haris, et al. in a small study have shown that T1rho MRI (a technique that can probe the protein content of various tissues) may be useful in the early diagnosis of AD.^41^

**Vascular MRI**

T2-weighted and fluid-attenuated inversion-recovery (FLAIR) images are used to identify amyloid-related imaging abnormalities (ARIAs, vasogenic edema and microhemorrhages) which are associated with Aβ-lowering drugs.^42^ Regulatory authorities require usage of vascular MRI for safety reasons in clinical trials using DMT. The occurrence of ARIAs is dependent on the dose of anti-Aβ drug and ApoEε4 genotype.^43^

**Diffusion tensor imaging (DTI) MRI**

DTI is based on the directionality of diffusion in the brain parenchyma and is used to assess white matter orientation and integrity using two parameters: fractional anisotropy (a marker of axonal integrity and myelination) and mean diffusivity (marker of cellular integrity). DTI can supplement volumetric MRI by depicting characteristic disruptions in neuronal connections.^44^

**Functional MRI (fMRI)**

As the name suggests fMRI provides an insight into cerebral functioning. In this modality, statistical maps of cerebral activation are produced based on changes in regional microcirculation inferred from measuring changes in blood-oxygen-level dependent (BOLD) MR signal. The MRI signal changes because of changes in blood flow, volume and oxyhemoglobin/deoxyhemoglobin ratio induced by external stimuli, specific tasks or drugs. Decreased activity in the hippocampus/MTL and increased activity in the prefrontal cortex is seen during encoding of new information in patients with AD and prodromal AD.^45^^,^^46^

Increased activity may be sometimes seen in the MTL in the early stages of the disease and in individuals at genetic risk of AD, and this has been attributed to compensatory mechanisms during hippocampal failure.^47^ Apart from the MTL, memory function is also subserved by the “default mode network (DMN)” (precuneus, posterior cingulate, lateral parietal, lateral temporal and medial prefrontal regions).^48^ The DMN that normally exhibits beneficial deactivation in healthy subjects, shows increased activity in both preclinical and clinical AD patients.^47^^-^^50^

**Task-free resting-state fMRI (rs-fMRI)**

It is more easily applicable and places less technical demands than activation-task fMRI, especially in severely demented patients. Impaired DMN has been shown even on rs-fMRI and with a direct correlation to disease severity. rs-fMRI has been found to be a stronger classifier than activation-task fMRI in distinguishing risk groups in non-demented adults carrying familial AD genes.^49^

**Arterial spin labeling (ASL) MRI**

This is an fMRI technique for measuring tissue perfusion using magnetically labelled protons in blood as an endogenous contrast agent. ASL MRI in comparison with perfusion PET was found to be as informative about regional hypoperfusion in prodromal AD and symptomatic AD, with greater resolution and no radiation exposure.^51^^,^^52^ Although this modality is promising, standardization issues need to be addressed. ^18^

**Fluoro-2-deoxy-D-glucose (FDG) positron emission tomography (PET)**

PET involves detection of two oppositely directed annihilation photons generated by positron-emitting radiopharmaceuticals. FDG is a glucose analogue and enters cells by the same transport mechanism as glucose and is phosphorylated to FDG-6-phosphate (FDG-6-P). FDG-6-P does not enter into further enzymatic pathways and accumulates in the intracellular compartment proportional to the glycolytic rate of the cell. While FDG-PET is considered mainly a measure of synaptic activity, BOLD fMRI is indicative of integrated neuronal synaptic activity. The pattern of resting FDG hypometabolism in AD involving the limbic and association areas has been called an “endophenotype” of AD. These hypometabolic areas are highly vulnerable to Aβ deposition and the FDG pattern correlates with histopathology at autopsy.^53^ The extent and severity of FDG hypometabolism is predictive of conversion of prodromal AD to AD, and directly correlates with cognitive decline.^54^^,^^55^

**Amyloid PET**

Amyloid PET involves use of Aβ-selective radioligands that bind to fibrillar Aβ.^18^ F-FDDNP was the first PET tracer to be used in AD. Although there was higher retention of the tracer in the hippocampus, amygdala and entorhinal cortex of AD patients, it had a relatively high non-specific binding and it also bound tau.^56^ The most commonly used tracer has been ^11^C-PiB (Pittsburgh Compound-B). However newer tracers that do not require a cyclotron like ^18^F-PiB and florbetaben, florbetapir and flutemetamol are being studied. In positive cases, Aβ deposits in a distribution that follows that of elevated aerobic glycolysis in the resting brain.^57^^,^^58^ Changes in amyloid PET can be seen as early as changes in CSF Aβ and so both may be used as screening tools, but as CSF Aβ reaches a final level early, amyloid PET is better at detecting cerebral amyloid load.^53^ The requirement of assessing disease progression is better served by structural MRI and FDG-PET vis-à-vis amyloid PET as amyloid deposition is an early event accumulating rapidly in the early stages and very slowly in the later stages.^53^^,^^56^ A direct correlation has been found between APOEε4 and Aβ deposition (as measured with ^11^C-PiB) and on CSF Aβ_1-42,_ but not on tau or p-tau_181_ levels, suggesting that Aβ initiates the disease.^56^ Aβ selective radioligands have also been used to provide evidence of dose-dependent reduction of Aβ PET signal on treatment with bapineuzumab and gantenerumab; this was however not associated with clinical improvement.^42^^,^^59^ Another development has been development of tracers directed at NFT: ^18^F-T-807, ^11^C-PBB-3.^60^^,^^61^

**Plasma protein biomarkers**

MRI and PET are expensive investigations and CSF studies are invasive, consequently influencing repeatability. Plasma biomarkers have the potential to be easily accessible and cheap markers of disease status. Hye, et al. proposed a panel of plasma proteins as biomarkers that could be used to predict progression of mild cognitive impairment to AD [transthyretin, CLU, cystatin C, A1 acid glycoprotein, complement C4, intercellular adhesion molecule (ICAM)-1, pigment epithelium-derived factor (PEDF), alpha-1 antitrypsin, regulated on activation normal T-cell expressed and secreted (RANTES), apolipoprotein C3] with an accuracy of 87 % as well as proteins associated with greater atrophy [alpha-1 antitrypsin, neuron specific enolase, brain derived neurotrophic factor (BDNF), apolipoproteins (C3, A1, E)] in AD.^62^ However, these proteins need to be validated in more longitudinal studies and the issue of specificity addressed as many of these proteins may not be specific to AD. Nevertheless, the concept of a suitable panel of plasma protein and imaging biomarkers for early diagnosis and monitoring progress of AD appears promising. 

These biomarkers are summarized in a tabular form in table 1.

**Targets for DMT**

The various targets of novel DMT can be broadly classified as a) interventions related to Aβ production, aggregation and clearance, b) immunotherapy against Aβ, and c) interventions related to tau hyperphosphorylation.^33^

**Interventions related to reduction of Aβ1-42 production, reduction of aggregation and increased clearance**

BACE-1 is modified by bisecting N-acetylglucosamine (GlcNAc) and AD patients have increased bisecting GlcNAc on BACE-1. Kizuka, et al. have shown that deficiency of GlcNAc-transferase (GnT)-III, the biosynthetic enzyme for GlcNAc, reduces cleavage of Aβ by BACE-1 resulting in reduced Aβ plaques and improved cognitive function in animal models.^63^ Thus, GnT-III and notch-sparing 2^nd^ generation γ-secretase inhibitors (e.g. begacestat, avagacestat, PF-3804014 and NIC5-15) are promising candidates for DMT.^64^ Aβ levels can also be reduced by blocking BACE-1 and trials are on anti-β secretase antibodies. Thiazolidinedione antidiabetic drugs (rosiglitazone, pioglitazone) via peroxisome proliferator-activated receptor-γ (PPAR-γ) activation can suppress BACE-1 expression. However, a lack of conclusive beneficial effects and the attendant cardiac risks have led to termination of rosiglitazone trials for AD.^65^ The results with pioglitazone in a pilot clinical trial have also been conflicting.^66^ Another strategy can be upregulation of α-secretase activity, leading to increased neuroprotective APPα. Though some drugs are undergoing trials, no results are yet available.

As the neurotoxic potential of Aβ oligomers is greater than Aβ monomers or fibrils, antiaggregants like ELND005 (scyllo-inositol), tramiprosate (homotaurine) and PBT2 are also under investigation.^67^ Though ELND005 did not achieve the trial endpoints, it did produce changes in CSF Aβ. Similarly, tramiprosate (binds soluble Aβ) and PBT2 (impedes metal-induced oligomerization of Aβ) did not achieve trial endpoints. 

Receptor for advanced glycation end-products (RAGE) mediates influx of Aβ and also mediates neuroinflammation and apoptosis, and low-density lipoprotein receptor-related protein 1 (LRP1) mediates efflux of Aβ from the brain. So their inhibitors and activators, respectively are targets of ongoing research.^68^^,^^69^ An oral RAGE-inhibitor (PF-04494700) has been tried but results were unsatisfactory.^70^ Another approach can be specific activation of proteases that degrade Aβ like neprilysin, insulin-degrading enzyme and plasmin. 

**Immunotherapy against Aβ**

Two approaches are being pursued: active immunity (anti-Aβ vaccine) using compounds containing the N-terminal fragment of Aβ_1-42 _or N-terminus mimic peptides (ACC-001, CAD106, V950, AFFITOPE^®^ AD02) and passive immunity using monoclonal anti-Aβ antibody (bapineuzumab, solanezumab).^71^^,^^72^ Though the clinical efficacy of bapineuzumab has not been consistent, it did demonstrate good tolerability with lowering of CSF p-tau and Aβ on PET. Solanezumab though not associated with ARIAs (amyloid-related imaging abnormalities), was also not found efficacious in mild to moderate AD.

**Table 1:** Biomarkers for Alzheimer’s disease (AD)

**Name**	**Advantages**		**Disadvantages**
APOEε4	Major genetic susceptibility factor for late-onset AD		Not diagnostic
	Carrying 1 allele increases risk factor by 2-3 times, 2 alleles increases the risk 16 times		Genetic studies are expensive
CSF Aβ1-42	Low Aβ_1-42 _is a sensitive marker of cerebral Aβ deposition	When Aβ1-42 and t-tau are considered together (Aβ1-42 to t-tau ratio), the sensitivity and specificity of diagnosing AD is more than 85 %	Invasive procedure
			Does not correlate well with duration or severity of disease
			Low CSF Aβ is not very specific for AD as it is also seen in other dementias
CSF tau	CSF tau (total-tau or t-tau and tau phosphorylated at threonine 181or p- tau 181) is increased in AD. Higher levels correlate with greater cognitive impairment		
	Different phosphorylated epitopes of tau maybe helpful in distinguishing AD and FTD (p-tau231) or AD and DLB (p-tau181)		
	p-tau/t-tau and p-tau/Aβ ratio are good indicators of severity of dementia and can differentiate between mild AD from moderate to severe AD		
CSF orexin	CSF orexin levels correlate with total tau protein levels, sleep impairment and cognitive decline in moderate to severe AD		Invasive procedure
			More studies required
MRI	Atrophy strongly related to dementia and closely matches clinical worsening in AD	Visual evaluation of MTL atrophy vis-à-vis normal ageing has a sensitivity and specificity of around 80%-85%	Expensive Not yet readily available
			More studies required and standardization issues need to be addressed
	DTI can supplement volumetric MRI by depicting characteristic disruptions in neuronal connections		
	Decreased activity in the hippocampus/MTL and increased activity in the prefrontal cortex is seen during encoding of new information in patients with AD and prodromal AD in Fmri		
	rs-fMRI has been found to be a stronger classifier than activation-task fMRI in distinguishing risk groups in non-demented adults carrying familial AD genes		
	T2-weighted and FLAIR images are used to identify ARIAs which are associated with Aβ-lowering drugs		
	ASL MRI in comparison with perfusion PET was found to be as informative about regional hypoperfusion in prodromal AD and symptomatic AD, with greater resolution and no radiation exposure		
FDG PET	The extent and severity of FDG hypometabolism is predictive of conversion of prodromal AD to AD, and directly correlates with cognitive decline		Expensive
			Not readily available
Amyloid PET	Changes in amyloid PET can be seen as early as changes in CSF Aβ and so both may be used as screening tools, but as CSF Aβ reaches a final level early, amyloid PET is better at detecting cerebral amyloid load		- The requirement of assessing disease progression is better served by structural MRI and FDG-PET vis-à-vis amyloid PET as amyloid deposition is an early event accumulating rapidly in the early stages and very slowly in the later stages
Plasma protein biomarkers	Potential to be easily accessible, cheap and repeatable		Need to be validated in more longitudinal studies
			Issues of specificity need to addressed as many of these proteins may not be specific to AD

AD: Alzheimer’s disease; MRI: Magnetic resonance imaging; DTI: Diffusion tensor imaging; APOEε4: Apolipoprotein E ε4; CSF: Cerebrospinal fluid; FTD: Frontotemporal dementia; DLB: Dementia of Lewy bodies; MTL: Medial temporal lobe; fMRI: Functional MRI; rs-fMRI: Resting-state functional MRI; FLAIR: Fluid-attenuated inversion-recovery; ARIAs: Amyloid-related imaging abnormalities; ASL: Arterial spin-labeling; FDG: ^18^Fluoro-2-deoxy-D-glucose; PET: Positron emission tomography

However, these drugs may still have a role in AD prevention. Recent studies have shown that passive immunity achieved by using intravenous immunoglobulin (IVIG) has an acceptable safety profile with encouraging changes in body fluid biomarkers.^73^^-^^75^

**Interventions targeted to tau hyperphosphorylation and aggregation**

Abnormally hyperphosphorylated and aggregated tau causes microtubule instability and axonal transport failure. Abnormal phosphorylation can be decreased by inhibiting tau kinases like glycogen synthase kinase (GSK)-3. GSK-3 inhibitors like valproic acid and lithium have not shown clinical efficacy or improvement in biomarkers. NP031112 (tideglusib), a non-ATP competitive GSK-3 inhibitor has been found to reduce p-tau and Aβ, prevent neuronal death and improve cognition in animals.^76^ Methylene blue (Rember™), an anti-tau aggregate and anti-oxidant has also been found to improve cognition in patients with mild to moderate AD.^77^

**Bioengineering and gene therapy**

Research is also underway in bioengineering techniques using stem cells and gene therapy to induce neurogenesis, angiogenesis, axonal regeneration and neuronal replacement by producing a milieu of Aβ degrading enzymes and neural growth factors.^78^

A number of studies from the Indian subcontinent have shown encouraging results from the use of medicinal herbs and antioxidants in AD in humans and animal models.^79^^-^^81^

The various approaches for novel DMT are summarized in table 2.

**Table 2:** Novel disease-modifying therapy (DMT)

**Class of drug/name**			**Mechanism of action**	**Special remarks**		
GnT-III and notch-sparing 2^nd^ generation γ-secretase inhibitors (e.g. begacestat, avagacestat, PF- 3804014 and NIC5-15)			BACE-1 is modified by bisecting GlcNAc and AD patients have increased bisecting GlcNAc on BACE-1			
	Deficiency of GnT-III, the biosynthetic enzyme for GlcNAc, reduces cleavage of Aβ by BACE-1 resulting in reduced Aβ plaques					
Anti-β secretase antibodies			Aβ levels can also be reduced by blocking BACE-1			
Thiazolidinedione antidiabetic drugs (rosiglitazone, pioglitazone)			Via PPAR-γ activation can suppress BACE-1 expression	Lack of conclusive beneficial effects and attendant cardiac risks have led to termination of rosiglitazone trials for AD		
Drugs upregulating α-secretase activity			Leads to increased neuroprotective APPα	Though some drugs are undergoing trials, no results are yet available		
Antiaggregants like ELND005 (scyllo-inositol), tramiprosate (homotaurine) and PBT2			Prevent aggregation of Aβ monomers as neurotoxic potential of oligomers is greater than Aβ monomers or fibrils	**-**		
Inhibitor for RAGE			RAGE mediates influx of Aβ and also mediates neuroinflammation and apoptosis	An oral RAGE-inhibitor (PF-04494700) has been tried but results were unsatisfactory		
Activators of receptor for LRP-1			Receptor for LRP-1 mediates efflux of Aβ from the brain	**-**		
Neprilysin, insulin-degrading enzyme and plasmin			Specific activation of proteases that degrade Aβ	**-**		
Active immunity (anti-Aβ vaccine, ACC-001, CAD106, V950, AFFITOPE^®^ AD02)			Using compounds containing the N- terminal fragment of Aβ1-42 or N- terminus mimic peptides	**-**		
Passive immunity (bapineuzumab, solanezumab)			Using monoclonal anti-Aβ antibody	**-**		
Tau kinase inhibitors			Abnormal phosphorylation can be decreased by inhibiting tau kinases like GSK-3	NP031112 (tideglusib), a non-ATP competitive GSK-3 inhibitor has been found to reduce p-tau and Aβ, prevent neuronal death and improve cognition in animals		
Methylene blue (Rember™)			Anti-tau aggregate and anti-oxidant	-		
Bioengineering techniques using stem cells and gene therapy		To induce neurogenesis, angiogenesis, axonal regeneration and neuronal replacement by producing a milieu of Aβ degrading enzymes and neural growth factors				-
Medicinal herbs and antioxidants in AD (black pepper, Padina gymnospora)			-	-		

GlcNAc: N-acetylglucosamine; GnT: GlcNAc-transferase; APPα: Amyloid precursor protein; BACE-1: B-site APP cleaving enzyme; AD: Alzheimer’s disease; RAGE: Receptor for advanced glycation end-products; LRP: low-density lipoprotein receptor-related protein 1; GSK: Glycogen synthase kinase; PPAR- γ: Peroxisome proliferator-activated receptor-γ

## Conclusion

We have come far in our understanding of the pathogenesis of AD with rapid strides being made in diagnostic modalities to detect the disease at a preclinical stage when novel DMT may be instituted to halt disease progression and also to monitor the effects of these drugs. However, more multi-institutional and longitudinal data is required to validate and standardize these modalities and select the most reliable, repeatable, easily available, cost-effective and complementary options. Synergizing the multipronged efforts of multiple bodies and institutions as represented by AD Neuroimaging Network (ADNI) and Dominantly Inherited Alzheimer Network (DIAN) are the way forward.

## Notes:

**How to cite this article:** Sen D, Majumder A, Arora V, Yadu N, Chakrabarti R. Taming Alzheimer’s disease: New perspectives, newer horizons. Iran J Neurol 2017; 16(3): 146-55.

## References

[1] Abbott A (2011). Dementia: A problem for our age. Nature.

[2] Selkoe DJ (2012). Preventing Alzheimer's disease. Science.

[3] Castro UR, Mulet JF, Claret I, Salarich J (1992). Thymus in posterior mediastinum. Cir Pediatr.

[4] Holtzman DM, Morris JC, Goate AM (2011). Alzheimer's disease: The challenge of the second century. Sci Transl Med.

[5] Small SA, Duff K (2008). Linking Abeta and tau in late-onset Alzheimer's disease: a dual pathway hypothesis. Neuron.

[6] Hyman BT (2011). Amyloid-dependent and amyloid-independent stages of Alzheimer disease. Arch Neurol.

[7] Hooper C, Killick R, Lovestone S (2008). The GSK3 hypothesis of Alzheimer's disease. J Neurochem.

[8] Karran E, Mercken M, De SB (2011). The amyloid cascade hypothesis for Alzheimer's disease: an appraisal for the development of therapeutics. Nat Rev Drug Discov.

[9] Iwatsubo T, Odaka A, Suzuki N, Mizusawa H, Nukina N, Ihara Y (1994). Visualization of A beta 42(43) and A beta 40 in senile plaques with end-specific A beta monoclonals: evidence that an initially deposited species is A beta 42(43). Neuron.

[10] Walsh DM, Klyubin I, Fadeeva JV, Cullen WK, Anwyl R, Wolfe MS (2002). Naturally secreted oligomers of amyloid beta protein potently inhibit hippocampal long-term potentiation in vivo. Nature.

[11] Chen Y, Neve R, Zheng H, Griffin W, Barger S, Mrak R (2014). Cycle on Wheels: Is APP Key to the AppBp1 Pathway?. Austin Alzheimers Parkinsons Dis.

[12] Corder EH, Saunders AM, Strittmatter WJ, Schmechel DE, Gaskell PC, Small GW (1993). Gene dose of apolipoprotein E type 4 allele and the risk of Alzheimer's disease in late onset families. Science.

[13] Farrer LA, Cupples LA, Haines JL, Hyman B, Kukull WA, Mayeux R (1997). Effects of age, sex, and ethnicity on the association between apolipoprotein E genotype and Alzheimer disease. A meta-analysis. APOE and Alzheimer Disease Meta-Analysis Consortium. JAMA.

[14] Price DL, Sisodia SS (1998). Mutant genes in familial Alzheimer's disease and transgenic models. Annu Rev Neurosci.

[15] Tanzi RE, Kovacs DM, Kim TW, Moir RD, Guenette SY, Wasco W (1996). The gene defects responsible for familial Alzheimer's disease. Neurobiol Dis.

[16] Griffin WS, Sheng JG, McKenzie JE, Royston MC, Gentleman SM, Brumback RA (1998). Life-long overexpression of S100beta in Down's syndrome: implications for Alzheimer pathogenesis. Neurobiol Aging.

[17] Levy-Lahad E, Wasco W, Poorkaj P, Romano DM, Oshima J, Pettingell WH (1995). Candidate gene for the chromosome 1 familial Alzheimer's disease locus. Science.

[18] Sherrington R, Rogaev EI, Liang Y, Rogaeva EA, Levesque G, Ikeda M (1995). Cloning of a gene bearing missense mutations in early-onset familial Alzheimer's disease. Nature.

[19] Braskie MN, Jahanshad N, Stein JL, Barysheva M, McMahon KL, de Zubicaray GI (2011). Common Alzheimer's disease risk variant within the CLU gene affects white matter microstructure in young adults. J Neurosci.

[20] Guerreiro R, Wojtas A, Bras J, Carrasquillo M, Rogaeva E, Majounie E (2013). TREM2 variants in Alzheimer's disease. N Engl J Med.

[21] Mawuenyega KG, Sigurdson W, Ovod V, Munsell L, Kasten T, Morris JC (2010). Decreased clearance of CNS beta-amyloid in Alzheimer's disease. Science.

[22] Ittner LM, Gotz J (2011). Amyloid-beta and tau--a toxic pas de deux in Alzheimer's disease. Nat Rev Neurosci.

[23] Ittner LM, Ke YD, Delerue F, Bi M, Gladbach A, van EJ (2010). Dendritic function of tau mediates amyloid-beta toxicity in Alzheimer's disease mouse models. Cell.

[24] Marquer C, Laine J, Dauphinot L, Hanbouch L, Lemercier-Neuillet C, Pierrot N (2014). Increasing membrane cholesterol of neurons in culture recapitulates Alzheimer's disease early phenotypes. Mol Neurodegener.

[25] Casoli T, Di SG, Spazzafumo L, Balietti M, Giorgetti B, Giuli C (2014). Contribution of non-reference alleles in mtDNA of Alzheimer's disease patients. Ann Clin Transl Neurol.

[26] Beck TN, Nicolas E, Kopp MC, Golemis EA (2014). Adaptors for disorders of the brain? The cancer signaling proteins NEDD9, CASS4, and PTK2B in Alzheimer's disease. Oncoscience.

[27] Jahangir Z, Ahmad W, Shabbiri K (2014). Alternate Phosphorylation/O-GlcNAc Modification on Human Insulin IRSs: A Road towards Impaired Insulin Signaling in Alzheimer and Diabetes. Adv Bioinformatics.

[28] Faldu KG, Shah JS, Patel SS (2015). Anti-Viral Agents in Neurodegenerative Disorders: New Paradigm for Targeting Alzheimer's Disease. Recent Pat Antiinfect Drug Discov.

[29] Mohajeri MH, Troesch B, Weber P (2015). Inadequate supply of vitamins and DHA in the elderly: implications for brain aging and Alzheimer-type dementia. Nutrition.

[30] Roe CM, Mintun MA, D'Angelo G, Xiong C, Grant EA, Morris JC (2008). Alzheimer disease and cognitive reserve: variation of education effect with carbon 11-labeled Pittsburgh Compound B uptake. Arch Neurol.

[31] Fotenos AF, Mintun MA, Snyder AZ, Morris JC, Buckner RL (2008). Brain volume decline in aging: evidence for a relation between socioeconomic status, preclinical Alzheimer disease, and reserve. Arch Neurol.

[32] Liang KY, Mintun MA, Fagan AM, Goate AM, Bugg JM, Holtzman DM (2010). Exercise and Alzheimer's disease biomarkers in cognitively normal older adults. Ann Neurol.

[33] Kang JH, Ryoo NY, Shin DW, Trojanowski JQ, Shaw LM (2014). Role of cerebrospinal fluid biomarkers in clinical trials for Alzheimer's disease modifying therapies. Korean J Physiol Pharmacol.

[34] Huded CB, Bharath S, Chandra SR, Sivakumar PT, Varghese M, Subramanian S (2015). Supportive CSF biomarker evidence to enhance the National Institute on Aging-Alzheimer's Association criteria for diagnosis of Alzheimer's type dementia--a study from Southern India. Asian J Psychiatr.

[35] Hampel H, Buerger K, Zinkowski R, Teipel SJ, Goernitz A, Andreasen N (2004). Measurement of phosphorylated tau epitopes in the differential diagnosis of Alzheimer disease: a comparative cerebrospinal fluid study. Arch Gen Psychiatry.

[36] Buerger K, Zinkowski R, Teipel SJ, Tapiola T, Arai H, Blennow K (2002). Differential diagnosis of Alzheimer disease with cerebrospinal fluid levels of tau protein phosphorylated at threonine 231. Arch Neurol.

[37] Liguori C, Romigi A, Nuccetelli M, Zannino S, Sancesario G, Martorana A (2014). Orexinergic system dysregulation, sleep impairment, and cognitive decline in Alzheimer disease. JAMA Neurol.

[38] Merlo PE, Jeromin A, Frisoni GB, Hill D, Lockhart A, Schmidt ME (2014). Imaging as a biomarker in drug discovery for Alzheimer's disease: Is MRI a suitable technology?. Alzheimers Res Ther.

[39] Savva GM, Wharton SB, Ince PG, Forster G, Matthews FE, Brayne C (2009). Age, neuropathology, and dementia. N Engl J Med.

[40] Frisoni GB, Fox NC, Jack CR Jr, Scheltens P, Thompson PM (2010). The clinical use of structural MRI in Alzheimer disease. Nat Rev Neurol.

[41] Haris M, Yadav SK, Rizwan A, Singh A, Cai K, Kaura D (2015). T1rho MRI and CSF biomarkers in diagnosis of Alzheimer's disease. Neuroimage Clin.

[42] Sperling R, Salloway S, Brooks DJ, Tampieri D, Barakos J, Fox NC (2012). Amyloid-related imaging abnormalities in patients with Alzheimer's disease treated with bapineuzumab: a retrospective analysis. Lancet Neurol.

[43] Sperling RA, Bates JF, Chua EF, Cocchiarella AJ, Rentz DM, Rosen BR (2003). fMRI studies of associative encoding in young and elderly controls and mild Alzheimer's disease. J Neurol Neurosurg Psychiatry.

[44] Sole-Padulles C, Bartres-Faz D, Junque C, Vendrell P, Rami L, Clemente IC (2009). Brain structure and function related to cognitive reserve variables in normal aging, mild cognitive impairment and Alzheimer's disease. Neurobiol Aging.

[45] Johnson SC, Schmitz TW, Moritz CH, Meyerand ME, Rowley HA, Alexander AL (2006). Activation of brain regions vulnerable to Alzheimer's disease: the effect of mild cognitive impairment. Neurobiol Aging.

[46] Raichle ME, MacLeod AM, Snyder AZ, Powers WJ, Gusnard DA, Shulman GL (2001). A default mode of brain function. Proc Natl Acad Sci USA.

[47] Daselaar SM, Prince SE, Cabeza R (2004). When less means more: deactivations during encoding that predict subsequent memory. Neuroimage.

[48] Miller SL, Celone K, DePeau K, Diamond E, Dickerson BC, Rentz D (2008). Age-related memory impairment associated with loss of parietal deactivation but preserved hippocampal activation. Proc Natl Acad Sci USA.

[49] Fleisher AS, Sherzai A, Taylor C, Langbaum JB, Chen K, Buxton RB (2009). Resting-state BOLD networks versus task-associated functional MRI for distinguishing Alzheimer's disease risk groups. Neuroimage.

[50] Sperling RA, Dickerson BC, Pihlajamaki M, Vannini P, LaViolette PS, Vitolo OV (2010). Functional alterations in memory networks in early Alzheimer's disease. Neuromolecular Med.

[51] Binnewijzend MA, Kuijer JP, Benedictus MR, van der Flier WM, Wink AM, Wattjes MP (2013). Cerebral blood flow measured with 3D pseudocontinuous arterial spin-labeling MR imaging in Alzheimer disease and mild cognitive impairment: a marker for disease severity. Radiology.

[52] Wolk DA, Detre JA (2012). Arterial spin labeling MRI: an emerging biomarker for Alzheimer's disease and other neurodegenerative conditions. Curr Opin Neurol.

[53] Johnson KA, Fox NC, Sperling RA, Klunk WE (2012). Brain imaging in Alzheimer disease. Cold Spring Harb Perspect Med.

[54] Reiman EM (2011). Fluorodeoxyglucose positron emission tomography: emerging roles in the evaluation of putative Alzheimer's disease-modifying treatments. Neurobiol Aging.

[55] Dukart J, Kherif F, Mueller K, Adaszewski S, Schroeter ML, Frackowiak RS (2013). Generative FDG-PET and MRI model of aging and disease progression in Alzheimer's disease. PLoS Comput Biol.

[56] Vlassenko AG, Benzinger TL, Morris JC (2012). PET amyloid-beta imaging in preclinical Alzheimer's disease. Biochim Biophys Acta.

[57] Vlassenko AG, Vaishnavi SN, Couture L, Sacco D, Shannon BJ, Mach RH (2010). Spatial correlation between brain aerobic glycolysis and amyloid-beta (Abeta) deposition. Proc Natl Acad Sci USA.

[58] Vaishnavi SN, Vlassenko AG, Rundle MM, Snyder AZ, Mintun MA, Raichle ME (2010). Regional aerobic glycolysis in the human brain. Proc Natl Acad Sci USA.

[59] Ostrowitzki S, Deptula D, Thurfjell L, Barkhof F, Bohrmann B, Brooks DJ (2012). Mechanism of amyloid removal in patients with Alzheimer disease treated with gantenerumab. Arch Neurol.

[60] Wood H (2013). Alzheimer disease: [11C]PBB3--a new PET ligand that identifies tau pathology in the brains of patients with AD. Nat Rev Neurol.

[61] Chien DT, Bahri S, Szardenings AK, Walsh JC, Mu F, Su MY (2013). Early clinical PET imaging results with the novel PHF-tau radioligand [F-18]-T807. J Alzheimers Dis.

[62] Hye A, Riddoch-Contreras J, Baird AL, Ashton NJ, Bazenet C, Leung R (2014). Plasma proteins predict conversion to dementia from prodromal disease. Alzheimers Dement.

[63] Kizuka Y, Kitazume S, Fujinawa R, Saito T, Iwata N, Saido TC (2015). An aberrant sugar modification of BACE1 blocks its lysosomal targeting in Alzheimer's disease. EMBO Mol Med.

[64] Tong G, Wang JS, Sverdlov O, Huang SP, Slemmon R, Croop R (2012). Multicenter, randomized, double-blind, placebo-controlled, single-ascending dose study of the oral gamma-secretase inhibitor BMS-708163 (Avagacestat): Tolerability profile, pharmacokinetic parameters, and pharmacodynamic markers. Clin Ther.

[65] Harrington C, Sawchak S, Chiang C, Davies J, Donovan C, Saunders AM (2011). Rosiglitazone does not improve cognition or global function when used as adjunctive therapy to AChE inhibitors in mild-to-moderate Alzheimer's disease: Two phase 3 studies. Curr Alzheimer Res.

[66] Geldmacher DS, Fritsch T, McClendon MJ, Landreth G (2011). A randomized pilot clinical trial of the safety of pioglitazone in treatment of patients with Alzheimer disease. Arch Neurol.

[67] Salloway S, Sperling R, Keren R, Porsteinsson AP, van Dyck CH, Tariot PN (2011). A phase 2 randomized trial of ELND005, scyllo-inositol, in mild to moderate Alzheimer disease. Neurology.

[68] Shibata M, Yamada S, Kumar SR, Calero M, Bading J, Frangione B (2000). Clearance of Alzheimer's amyloid-ss(1-40) peptide from brain by LDL receptor-related protein-1 at the blood-brain barrier. J Clin Invest.

[69] Deane R, Du YS, Submamaryan RK, LaRue B, Jovanovic S, Hogg E (2003). RAGE mediates amyloid-beta peptide transport across the blood-brain barrier and accumulation in brain. Nat Med.

[70] Galasko D, Bell J, Mancuso JY, Kupiec JW, Sabbagh MN, van, Dyck C (2014). Clinical trial of an inhibitor of RAGE-Abeta interactions in Alzheimer disease. Neurology.

[71] Winblad B, Andreasen N, Minthon L, Floesser A, Imbert G, Dumortier T (2012). Safety, tolerability, and antibody response of active Abeta immunotherapy with CAD106 in patients with Alzheimer's disease: randomised, double-blind, placebo-controlled, first-in-human study. Lancet Neurol.

[72] Salloway S, Sperling R, Fox NC, Blennow K, Klunk W, Raskind M (2014). Two phase 3 trials of bapineuzumab in mild-to-moderate Alzheimer's disease. N Engl J Med.

[73] Farlow M, Arnold SE, van Dyck CH, Aisen PS, Snider BJ, Porsteinsson AP (2012). Safety and biomarker effects of solanezumab in patients with Alzheimer's disease. Alzheimers Dement.

[74] Dodel R, Rominger A, Bartenstein P, Barkhof F, Blennow K, Forster S (2013). Intravenous immunoglobulin for treatment of mild-to-moderate Alzheimer's disease: a phase 2, randomised, double-blind, placebo-controlled, dose-finding trial. Lancet Neurol.

[75] Knight EM, Gandy S (2014). Immunomodulation and AD--down but not out. J Clin Immunol.

[76] Sereno L, Coma M, Rodriguez M, Sanchez-Ferrer P, Sanchez MB, Gich I (2009). A novel GSK-3beta inhibitor reduces Alzheimer's pathology and rescues neuronal loss in vivo. Neurobiol Dis.

[77] Wischik CM, Bentham P, Wischik DJ, Seng KM (2008). Tau aggregation inhibitor (TAI) therapy with rember™ arrests disease progression in mild and moderate Alzheimer's disease over 50 weeks. Alzheimers Dement.

[78] Huang C, Chu H, Muheremu A, Zuo H (2015). Neurorestorative strategies for Alzheimer's disease. Neurol India.

[79] Nishteswar K, Joshi H, Karra RD (2014). Role of indigenous herbs in the management of Alzheimer's disease. Anc Sci Life.

[80] Subedee L, Suresh RN, Mk J, Hl K, Am S, Vh P (2015). Preventive role of Indian black pepper in animal models of Alzheimer's disease. J Clin Diagn Res.

[81] Kumar A, Sharma V, Singh VP, Kaundal M, Gupta MK, Bariwal J (2015). Herbs to curb cyclic nucleotide phosphodiesterase and their potential role in Alzheimer's disease. Mech Ageing Dev.

